# PD-L1 promotes GSDMD-mediated NET release by maintaining the transcriptional activity of Stat3 in sepsis-associated encephalopathy

**DOI:** 10.7150/ijbs.79913

**Published:** 2023-02-27

**Authors:** Cheng-long Zhu, Jian Xie, Qiang Liu, Yi Wang, Hui-ru Li, Chang-meng Yu, Peng Li, Xiao-ming Deng, Jin-jun Bian, Jia-feng Wang

**Affiliations:** 1 Faculty of Anesthesiology, Changhai Hospital, Naval Medical University, Shanghai, People's Republic of China; 2 Jiangsu Province Key Laboratory of Anesthesiology, Xuzhou Medical University, Xuzhou, Jiangsu Province, People's Republic of China; 3 Faculty of Anesthesiology, Weifang Medical University, Weifang, Shandong Province, People's Republic of China

**Keywords:** sepsis-associated encephalopathy, neutrophil, neutrophil extracellular traps, PD-L1, gasdermin D

## Abstract

Sepsis-associated encephalopathy (SAE), as shown as acute and long-term cognitive impairment, is associated with increased mortality of sepsis. The causative factors of SAE are diverse and the underlying pathological mechanisms of SAE remain to be fully elucidated. Multiple studies have demonstrated a crucial role of microglia in the development of SAE, but the role of neutrophils and neutrophil extracellular traps (NETs) in SAE is still unclear. Here, we firstly show that in murine sepsis model, neutrophils and NETs promote blood-brain barrier (BBB) disruption, neuronal apoptosis and microglia activation in hippocampus and induce hippocampus-dependent memory impairment. Anti-Gr-1 antibody or DNase I treatment attenuates these sepsis-induced changes. Then, we find that genetic deletion of neutrophil GSDMD or PD-L1 reduces NET release and improves SAE in murine sepsis model. Finally, in human septic neutrophils, p-Y705-Stat3 binds to PD-L1, promotes PD-L1 nuclear translocation and enhances transcription of the gasdermin D (GSDMD) gene. In summary, our findings firstly identify a novel function of PD-L1 in maintaining transcriptional activity of p-Y705-Stat3 to promote GSDMD-dependent NET release in septic neutrophils, which plays a critical role in the development of SAE.

## Introduction

Sepsis is an overwhelming response to infection and defined as a life-threatening multi-organ dysfunction with an extraordinarily high mortality rate 1, 2. A recent survey showed that around 48.9 million individuals globally suffered from sepsis each year, and 11 million sepsis-related deaths might occur 3. Multiple organ dysfunction may be present during sepsis, including the central nervous system (CNS), and the brain is regarded as one of the first organs to be challenged to inflammation 4, 5. Sepsis-associated encephalopathy (SAE) refers to a widespread CNS dysfunction, which is not caused by a direct CNS infection 6, 7. More than half of the septic patients have symptoms of SAE, and mortality rises with the severity of SAE, reaching 70% in certain cases 5, 8. SAE manifests as a series of brain disorders that interfere with life, and the pathophysiological mechanism of SAE is still unclear. The mortality of sepsis increases with the severity of SAE, so it is important to investigate the pathogenesis of SAE to identify a therapeutic target to reduce the related morbidity and mortality 5, 9, 10.

Neutrophils are a double-edged sword, serving as both an essential initial line of defense against invading pathogens and, in certain cases, a mediator of tissue destruction 11-13. In addition to the dual role of neutrophils, there is a similar dual role for NETs released by neutrophil activation, which are extensive web-like DNA structures containing neutrophil nuclear and granular contents, including double-stranded DNA, histone, neutrophil elastase (NE), myeloperoxidase (MPO) and cathepsin G 14. NETs were initially shown to play a protective role for the host, but subsequently an increasing number of studies have shown that NETs also mediate tissue damage under various inflammatory conditions, including sepsis 14-18. NETs have been reported to be associated with a variety of diseases including autoimmune diseases 19, inflammation 20, thrombosis 21, cardiovascular disease 22, and lung disease 23, 24. Importantly, neutrophils and release of NETs have been found in the brain of animal models of SAE 10, 25. However, the role of neutrophils and NETs in the pathogenesis of SAE has not been well established.

In sepsis, pore-forming protein gasdermin-D (GSDMD) is closely related to the release of inflammatory factors and immune dysregulation, and inhibition of GSDMD improves the survival rate of septic animals 26. It has been also reported that GSDMD can be cleaved in neutrophils from septic mice and humans, playing a crucial role in NET release and organ dysfunction during sepsis 27-29. Neutrophil GSDMD-mediated NETs promote sepsis-related lung, heart, kidney, and liver injury, whereas inhibiting GSDMD reverses the sepsis-induced damage in these organs 27, but the role of neutrophil GSDMD in SAE has not been identified.

We previously reported that the critical immune checkpoint programmed death ligand 1 (PD-L1) was up-regulated on neutrophils in sepsis, PD-L1^+^ neutrophil could transmit an inhibitory signal by exerting immune checkpoint function to induce T-lymphocyte apoptosis and immunosuppression 30. We also found that neutrophil PD-L1 had non-immune-checkpoint function. PD-L1 inhibited the spontaneous apoptotic response of septic neutrophils and led to excessive neutrophil accumulation to cause lung injury in septic mice 31. Moreover, NET release was also promoted by PD-L1 in septic lung injury in our previous study 24, while the exact mechanism of PD-L1 in regulating NET release was not well described.

Therefore, the present study was performed to identify the role and the related mechanisms of neutrophils and NETs in SAE. Whether GSDMD and PD-L1 were involved in the NET release and development of encephalopathy was also investigated. Moreover, we demonstrated whether there was a link between PD-L1 and GSDMD in regulating the NET release.

## Methods

### Study Design

The aim of this study was to determine the relationship between PD-L1 and GSDMD-mediated NET release in septic neutrophils and the impact on SAE. To do this, we observed GSDMD expression and NET formation after PD-L1 knockout in human septic neutrophils *in vitro* and investigated the role of p-Y705-Stat3 in PD-L1 translocation to the nucleus. GSDMD and PD-L1 CKO mice were used to study the effect of neutrophil NETs on the development of SAE. When image quantification required manual image scoring, images were blinded and randomized prior to analysis by researchers not involved in image acquisition. Randomization was used in CLP modeling.

### Patients

We recruited patients with sepsis who met the clinical criteria of sepsis 3.0 definition 32 from the central intensive care unit of Shanghai Changhai Hospital, China, and healthy volunteers were recruited as controls. Peripheral blood was taken from patients within the first 24 hours after admission to ICU. Neutrophils are then extracted within one hour. The study protocol was approved by the Committee on Ethics of Biomedicine Research in Naval Medical University, Shanghai, China.

### Mice

C57BL/6J mice (male, 8-10 weeks old) were purchased from GemPharmatech Laboratory Animal Company (Nanjing, China). Neutrophil specific PD-L1 or GSDMD conditional knockout (CKO) mice were generated by crossing PD-L1^flox/flox^ or GSDMD^flox/flox^, engineered using CRISPR/Cas9 (BIORAY LABORATORIES Inc., Shanghai, China), with elane (Ela)^cre/cre^ mice 31, 33, 34 purchased from the EMMA mouse repository (INFRAFRONTIER, Munchen, Germany). Neutrophil PD-L1 CKO mice were used in our previous study 24, 31. The detailed information about neutrophil GSDMD CKO mice can be found in our previous study 35. All mice were under a 12 h light/12 h dark schedule (lights on at 7:00, off at 19:00), with free access to water and food. For the studies, littermate controls (Ela^cre^-PD-L1^WT^ or Ela^cre^-GSDMD^WT^) were employed, and mice were divided into control and experimental groups. All animal studies were approved by the Committee on Ethics of Biomedicine Research in Naval Medical University, Shanghai, China.

### Neutrophil purification

As previously reported 36, human neutrophils were purified by density gradient centrifugation with 3% Dextran and Ficoll-Hypaque solution (17-1440-03, GE Healthcare, Little Chalfont, UK). Mice blood neutrophils were isolated by positive selection magnetic cell separation (MACS) using the Neutrophil Isolation Kit (130-097-658, Miltenyi Biotec, Bergisch Gladbach, Germany) according to instructions. At a concentration of 1 x 10^6^ cells/ml, the PMN pellet was resuspended in DMEM supplemented with 10% FBS, 1% glutamine, and 1% penicillin/streptomycin solution.

### Cecal ligation and puncture (CLP) model

Mice were randomly assigned to either cecal ligation and puncture (CLP) surgery or sham surgery using a randomization table. The CLP model was performed as described previously 37. To induce CLP, sevoflurane-inhalation anesthesia was applied and the mice were punctured via the cecum with a 21-gauge needle. The cecum was exposed in sham-operated animals using a similar procedure, but without the ligation and puncture. Following surgery, all mice were given prewarmed normal saline (37°C; 5 ml per 100 g body weight) and tramadol (20mg/kg body weight) subcutaneously for postoperative resuscitation and analgesia. After waking up from anesthesia, all of the experimental mice had full access to food and drink.

### Neutrophil depletion

To deplete neutrophil, mice were treated with intraperitoneal (i.p.) injection of 150 µl (1 mg/kg) anti-Gr-1 antibody (Clone RB6-8C5, BE0075, BioXcell, NH) to deplete neutrophils 24 hours prior to the CLP surgery 38. To verify neutrophil depletion, blood neutrophil levels were assessed by flow cytometry operated on FACSCanto II flow cytometer (BD Bioscience, San Jose, CA, USA) with APC-conjugated anti-mouse Ly6G antibody (127613, BioLegend, San Diego, California, USA) and FITC-conjugated anti-mouse CD11b (101205, BioLegend, San Diego, California, USA). The data were analyzed with FlowJo V7.6 software (Tree Star, Inc., Ashland, OR).

### DNase I treatment

Mice were injected with 10 µg i.v. DNase I (Deoxyribonuclease I human recombinant; enz-319-10000IU, ProSpec, Israel) at 2 hours and 12 hours after CLP surgery. Control mice were injected with vehicle (8.77 mg/mL sodium chloride and 0.15 mg/mL calcium chloride).

### Fear conditioning (FC) tests

A fear conditioning device (XR-XZ301, New Soft Information Technology, Shanghai, China) was used to evaluate the hippocampus-dependent memory 39. Briefly, acclimatization was conducted initially, which consisted of a single 3 minutes exposure to the new environment, followed by 30 seconds of tone (65 dB, 3 kHz) and a single electric shock to the foot (3 s, 0.75 mA). 24 hours later, the context and tone fear conditioning tests were conducted. Mice were put in a room with a different form, color, and odor from the prior one two hours following the contextual fear conditioning test. To test the tone fear conditioning reaction, the tone delivery was trained for 3 minutes. The lack of any observable bodily movement other than breathing was described as freezing behavior.

### Measurement of BBB permeability

The permeability of BBB was measured with Evans Blue dye as previously described 40. Evans Blue dye (4% in saline, 2 ml/kg; Sigma-Aldrich, St. Louis, MO, USA) was injected into the tail vein 2 hours before sacrifice. The residual dye in the blood vessels of the mice was washed with normal saline. Hippocampus samples were then collected at 24 hours after CLP, weighed, and homogenized in PBS. The tissue homogenate was incubated in formamide at 60°C for 18 hours and Evans Blue was extracted. The amount of infiltrated Evans Blue was quantified by spectrophotometer at 620 nm. Data were expressed as micrograms per gram of hippocampus tissue weight.

### Small interfering RNA transfection

According to the manufacturer's instructions, human neutrophils were transiently transfected with PD-L1 siRNA (sc-39699) or non-targeting siRNA (sc-37007) as a control siRNA (Santa Cruz Inc, CA, USA). Briefly, 1×10^6^ cells were incubated in DMEM with 10% FBS, 1% glutamine, and 1% penicillin/streptomycin solution at a concentration of 1×10^6^ cells/ml, and then transfected with the indicated siRNAs using Lipofectamine (Invitrogen, Carlsbad, CA, USA) according to the manufacturer's instructions. The impact of knockdown on PD-L1 protein expression was assessed by western blotting the next day, after 21 hours of recovery on full media.

### Cytoplasmic nuclear fractionation

According to the instructions, NE-PER Nuclear and Cytoplasmic Extraction Reagents (78833, Pierce, Rockford, IL, USA) were used to separate cytoplasmic and nuclear extracts, followed by protein isolation.

### Chromatin immunoprecipitation

Chromatin immunoprecipitation was performed using the ChIP kit (26156, Pierce, Rockford, IL, USA). p-Y705-Stat3-binding-site sequences in the GSDMD promoter were detected from the immunoprecipitants by PCR using primers listed in Table S1.

### Western Blotting

The hippocampus and neutrophil lysates were collected to conduct western immunoblotting. The denatured protein samples were separated by 10% SDS-PAGE gels and transferred to polyvinylidene fluoride (PVDF) membranes (Merck, Darmstadt, Merck, Germany). The transferred membranes were blocked with blocking solution (Thermo, Logan, UT, USA) and incubated with primary antibody overnight at 4°C. The HRP-conjugated secondary antibody (anti-rabbit/anti-mouse, AT0097/AT0098, 1:5000, Engibody Biotechnology, Milwaukee, WI, USA) was then added to the washed membrane and shaken for two hours at room temperature. Finally, we used the ECL reagent (Thermo, Massachusetts, USA) to visualize the membranes. The protein bands were analyzed by using ImageJ software. The primary antibodies included anti-Ly6G (1:1000, #87048, Cell Signaling Technology, Danvers, MA, USA), anti-Cit-H3 (1:1000, ab5103, Abcam, Massachusetts, USA), anti-PD-L1 (1:200, sc-518027, Santa Cruz, Dallas, TX, USA), anti-GSDMD (1:1000, ab210070, Abcam, Massachusetts, USA), anti-p-Y705-Stat3 (1:1000, #9145, Cell Signaling Technology, Danvers, MA, USA), anti-Stat3 (1:1000, #4904, Cell Signaling Technology, Danvers, MA, USA), anti-Lamin B1 (1:1000, #13435, Cell Signaling Technology, Danvers, MA, USA), anti-β-Tubulin (1:1000, #2128, Cell Signaling Technology, Danvers, MA, USA), and anti-β-actin (1:2000, AT0048, Engibody Biotechnology, Milwaukee, WI, USA).

### Immunoprecipitation

Immunoprecipitation (IP) buffer mixed with protease inhibitors was used to lyse the cells. The supernatants were rotated for 12 hours at 4°C treated with 2 μg of the relevant primary antibody (anti-p-Y705-Stat3, 1:1000, #9145, Cell Signaling Technology, Danvers, MA, USA). The supernatants were then rotated with Protein A/G agarose for 2 hours. The immunoprecipitants were washed three times with IP buffer before being eluted and boiled for 10 minutes with SDS sample solution. The immunoprecipitants were subsequently analyzed using western blots (anti-PD-L1, 1:200, sc-518027, Santa Cruz, Dallas, TX, USA).

### Immunofluorescence

Following anaesthetized, mice were perfused transcardially by phosphate-buffer saline (PBS) and 4% paraformaldehyde (PFA) successively. Brains were removed and fixed in PFA at 4°C for more than 24 hours. Thereafter, brains were immersed in 30% sucrose for dehydration and then cut into about 5-μm-thick slices on a cryostat. Tissue sections were incubated with 0.5% Triton X-100 for 20 minutes for permeation, and subsequently immersed in 3% bovine serum albumin (BSA) for 1 hour at room temperature for blocking. The sections were then incubated with primary antibodies (1:200, anti-IBA-1, 10904-1-AP, Proteintech, Illinois, USA; 1:200, anti-Cit-H3, ab5103, Abcam, Massachusetts, USA; 1:200, anti-MPO, ab208670, Abcam, Massachusetts, USA) overnight at 4°C. After washed with PBS for three times, the sections were incubated with secondary antibodies (1:1000) for 1 hour at room temperature. We used DAPI as a nuclear counterstain (Life Technologies).

For detecting mouse peripheral neutrophil GSDMD expression, isolated neutrophils were fixed with 4% PFA for 20 minutes. Next, neutrophils were permeabilized with 0.1% Triton X-100 and blocked with 10% normal donkey serum, 0.5% BSA, 2 mM EDTA and 0.02% TX-100 in PBS for 1 hour at 4°C. The slides were stained with anti-GSDMD (1:200, NBP2-33422, Novus, Colorado, USA). On the next day, the samples were incubated with secondary antibody (1:1000) for 1 hour. We used DAPI as a nuclear counterstain (Life Technologies).

After being rinsed and mounted with glycerol, the sections were recorded using fluorescence microscopy. Finally, the average number of positive IBA-1 stains is reflected as the percentage of the total image area that is positively stained in the average three fields, and ten sections of each group were analyzed. For calculating neutrophil GSDMD expression, the GSDMD expression intensity is reflected as GSDMD^+^ neutrophils/total neutrophils that are positively stained in the average three fields, and ten sections of each group were analyzed.

### Quantification of NETs in hippocampus

The NET scoring system was performed as described previously 41. Briefly, NETs were co-stained with MPO, Cit-H3 and DAPI. Then NETs were scored using established criteria based on two major morphologic patterns (individual strands or clusters). ImageJ software was used to calculate the area of clusters. The total score of hippocampus NETs was calculated as the sum of scores of 10 fields.

### TUNEL staining

We used TUNEL assay to determine apoptosis in the hippocampus using the *In situ* Death Detection Kit, AP (Roche, Basal, Switzerland). Hippocampus was taken 24 hours after operation and placed in 4% paraformaldehyde for fixation. After section preparation, the sections were dewaxed, hydrated, and treated with protease K. Sections were initially incubated with 3% hydrogen peroxide for 15 minutes. Subsequently, sections were incubated with deoxynucleotidyl transferase end reaction mixture at 37°C for 60 min, followed by DAPI incubation for 5 minutes. Fluorescence microscopy was performed to visualize TUNEL-positive cells (green-stained cells). Finally, the average number of positive TUNEL stains is reflected as the percentage of the total image area that is positively stained in the average three fields, and ten sections of each group were analyzed.

### Quantitative RT-PCR

Total RNA was extracted using the RNeasy mini kit (74104, Qiagen, Germany) as directed by the manufacturer, and then reverse transcription for first-strand cDNA synthesis was performed using the SuperScript III First-Strand Synthesis SuperMix (Invitrogen, Carlsbad, CA, USA). The primers that were used in quantitative PCR using the CFX96 touch real-time PCR detection system and the SYBR Green were listed in Table S1.

### Quantification of cell-free DNA

The levels of NET formation were measured using circulating free DNA (cf-DNA) 42. The Quant-iT Pico Green dsDNA assay (Invitrogen) was used to measure the levels of cf-DNA in the mouse plasma and supernatant of cultured neutrophils according to the manufacturer's instructions. A microplate reader was used to measure the fluorescence intensity, which represents the quantity of DNA, at an excitation wavelength of 485 nm and an emission wavelength of 530 nm (SpectraMax Gemini; Molecular Devices, Sunnyvale, CA). Serial dilutions of dsDNA (1-1,000 ng/ml) were used to create a standard curve.

### *In vitro* NET measure

The *in vitro* measurement of NET was performed as described previously 43. Primary neutrophils (2 × 10^6^ cells/ml) from sham-mice and CLP-mice, or septic patients and healthy volunteers were collected, washed with PBS for three times, fixed with 4% paraformaldehyde for 20 minutes, permeabilized with 0.1% Triton X-100 for 30 minutes at room temperature, and then blocked with 1% BSA in distilled water for 30 minutes. The cells were incubated overnight with an anti-Cit-H3 antibody (ab5103, Abcam, Massachusetts, USA) at a 1:500 dilution. After being washed with PBS three times, the cells were stained with secondary antibody: Alexa Fluor 488-conjugated anti-rabbit antibodies. DNA was stained with SYTOX Orange DNA fluorescent dyes. NET formation was identified as the percentage of the visual field positive for Cit-H3 signaling.

### Flow cytometry

Mice were euthanized 24 hours after CLP or sham-operated surgery to get blood. Cells were surface-stained with FITC-conjugated anti-mouse Ly6G antibody (127605, BioLegend, San Diego, California, USA), fixed, and permeabilized before intracellular staining with PE-conjugated anti-mouse GSDMD antibody (ab246713, Abcam, Massachusetts, USA) which pre-absorbed against cells overnight at 4°C. The stained cells were analyzed by flow cytometry operated on FACSCanto II flow cytometer (BD Bioscience, San Jose, CA, USA) and the data were analyzed using FlowJo V7.6 (Tree Star, Ashland, OR, USA).

### Statistical analysis

Graphpad Prism 8.0 (San Diego, CA, USA) was used for all statistical analyses. The continuous data were evaluated for distribution normality using Kolmogorov-Smirnov test. The 2-tailed Student's t test was used to compare two groups of normally distributed continuous data. One-way analysis of variance (ANOVA) was used to compare three or four groups of normally distributed continuous data, followed by a Tukey's test if needed. The mean and standard deviation (SD) was used to represent quantitative data. Statistically significant differences were shown as *p<0.05, **p<0.01, ***p<0.001, ****p<0.0001. ns=not significant.

## Results

### Neutrophils contribute to memory impairment and inflammatory damages of hippocampus in CLP-induced sepsis

**Figure 1:**
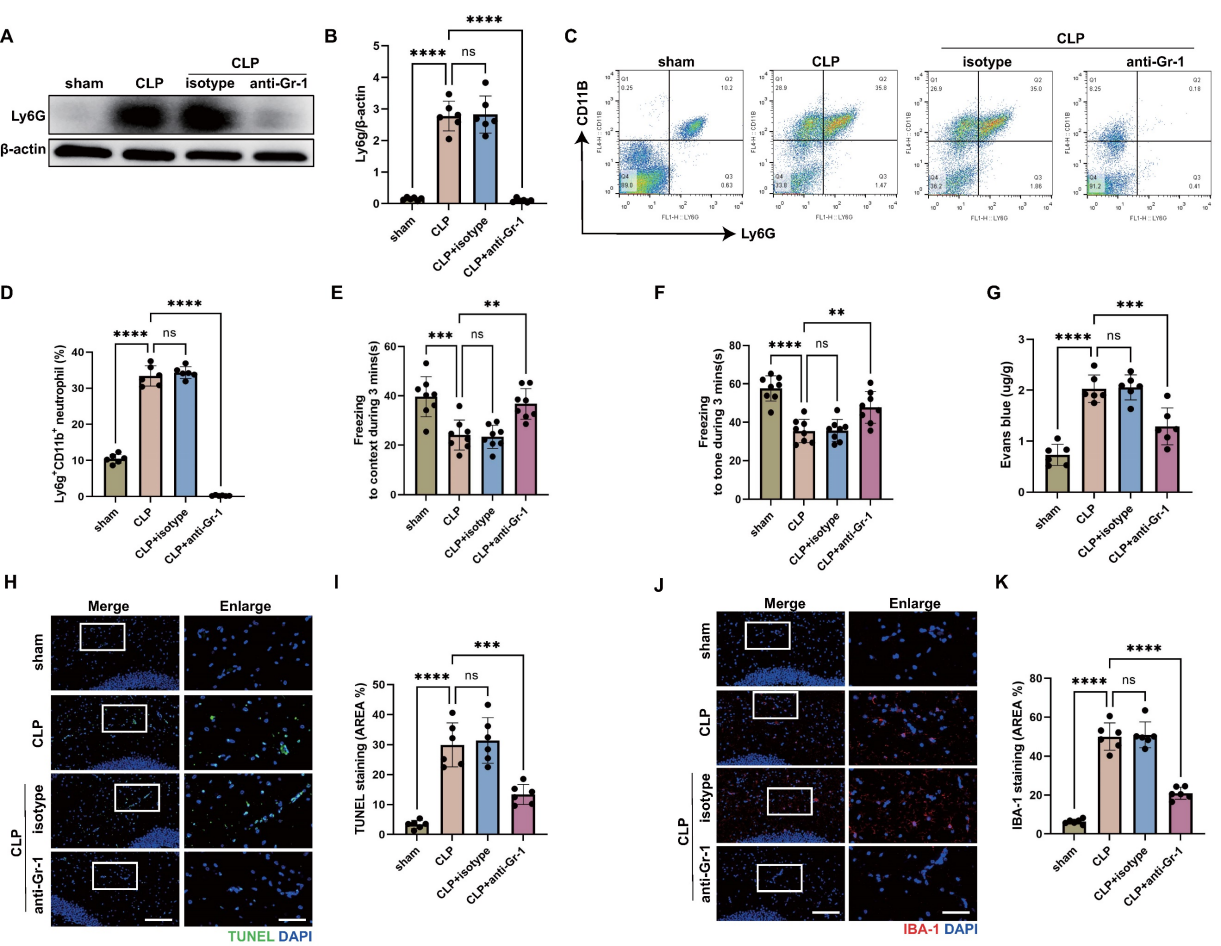
** Neutrophils accumulate in the hippocampus and contribute to the progression of SAE.** (A and B) Representative immunoblots and quantification of Ly6G levels in the hippocampus at 24 hours after operation. (C and D) Representative FACS plots and quantification of neutrophils (Ly6G^+^CD11b^+^) measured by flow cytometry in blood at 24 hours after operation. (E and F) Freezing to context and freezing to tone examined at 24 hours after operation. (G) The BBB permeability of hippocampus evaluated by Evans blue extravasation at 24 hours after operation. (H) Representative TUNEL (green) and DAPI (blue) immunofluorescence staining in the hippocampus. Scale bar indicates 20 μm. Higher magnification images are shown at the right row of figures-scale bar indicates 10 μm. (I) The quantitative results of the percentage of TUNEL-positive area in the total area of the image (whole microscopic field) in the hippocampus. (J) Representative IBA-1 (red) and DAPI (blue) immunofluorescence staining in the hippocampus. Scale bar indicates 20 μm. Higher magnification images are shown at the right row of figures-scale bar indicates 10 μm. (K) The quantitative results of the percentage of IBA-1 positive area in the total area of the image (whole microscopic field) in the hippocampus. The values are presented as mean ± SD (n=6 for Fig. B, D, G, I and K; n=8 for Fig. E and F; **P<0.01, ***P<0.001, ****P<0.0001, ns=not significant, one-way analysis of variance).

Increased infiltration of neutrophils in the central nervous system (CNS) has been reported at the early stage of sepsis 44. To investigate the role of neutrophils in sepsis-associated encephalopathy (SAE), we subjected wild type (WT) mice to SAE using cecal ligation and puncture (CLP) model and analyzed the brains at 24 hours after operation. Using an anti-lymphocyte antigen 6 complex locus G (Ly6G) antibody, we found elevated levels of neutrophils in the hippocampus of CLP mice (Fig. 1A-B). As reported, neutrophils accumulated in the CNS, damaging brain cells and playing an important role in the development of SAE 10. To verify the protective effect of neutrophil deletion on SAE, we administered anti-granulocyte receptor-1 (Gr-1) antibody to mice 24 hours before CLP operation to deplete neutrophils. The western blot for Ly6G in the hippocampus tissue and flow cytometry against Ly6G in the peripheral blood showed that neutrophils were almost completely depleted (Fig. 1A-D). Fear conditioning (FC) tests showed that CLP mice suffered from memory impairment at 24 hours after CLP surgery, whereas anti-Gr-1 antibody treatment ameliorated the memory dysfunction (Fig. 1E-F).

After a brain injury, leukocyte infiltration damages the blood-brain barrier (BBB) by releasing proinflammatory mediators, proteases, and reactive oxygen species (ROS) 45, 46. To investigate the role of neutrophils on BBB permeability of hippocampus during sepsis, Evans blue extravasation was performed. The results showed that CLP mice had a high BBB permeability in hippocampus, neutrophils depletion resulted in a significant improvement in the sepsis-induced increase of BBB permeability (Fig. 1G). In addition, neutrophils depletion attenuated neuronal apoptosis and suppressed the activation of microglia in the hippocampus of CLP mice (Fig. 1H-K). TUNEL assays showed that the rate of apoptotic cells in the hippocampus was greatly increased in CLP mice, while the situation was improved in anti-Gr-1-treated CLP mice (Fig. 1H-I). The fluorescence intensity of IBA-1 was significantly stronger in the CLP mice, while anti-Gr-1 treatment certainly reversed this change (Fig. 1J-K). Together, these results indicated that neutrophils could damage memory function and promote BBB disruption, neuronal apoptosis and microglia activation in hippocampus during sepsis.

### NETs are a key mediator of encephalopathy induced by sepsis

**Figure 2:**
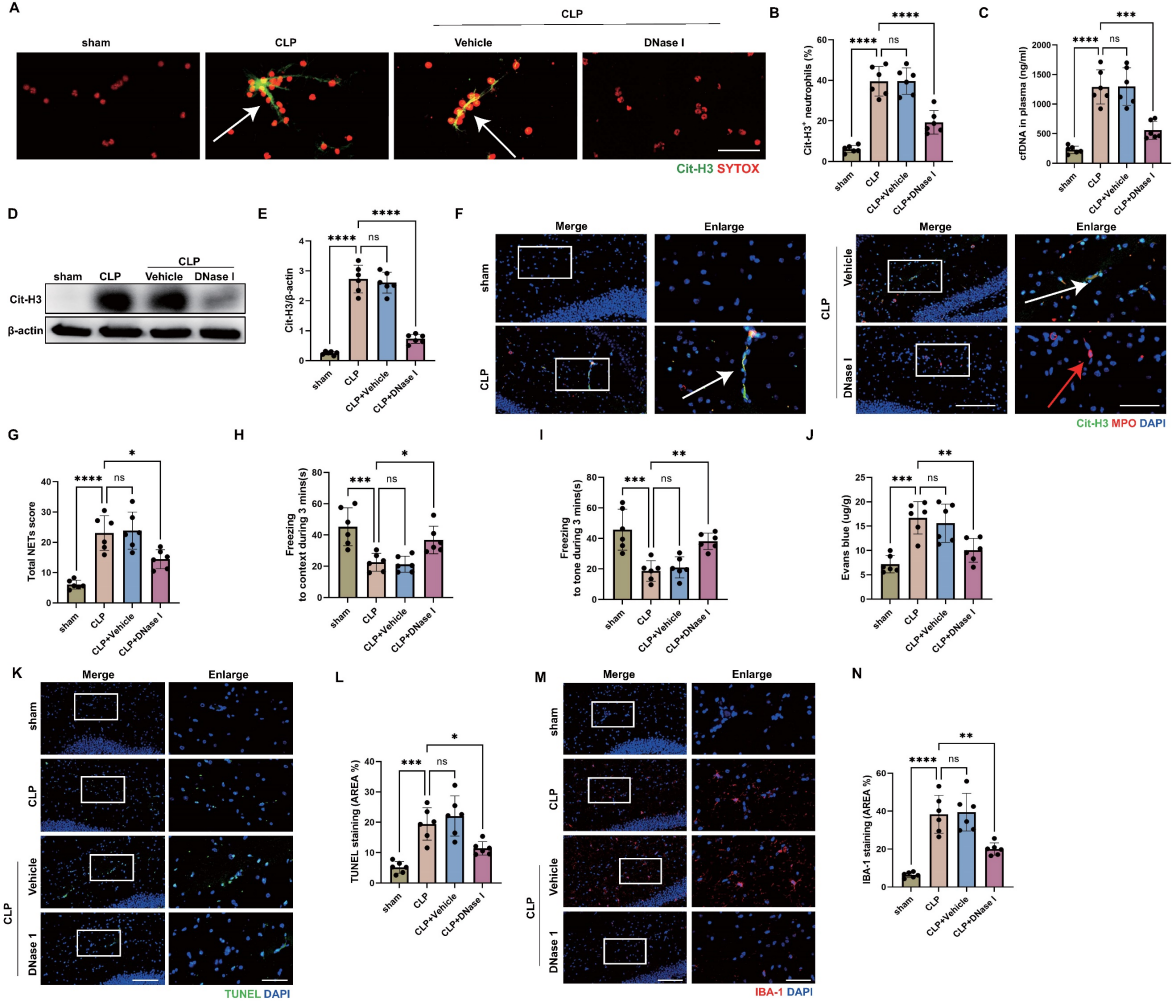
** Sepsis induces neutrophil releasing NETs and NETs digestion with DNase I attenuates the development of SAE.** (A) Representative immunofluorescence images of isolated peripheral blood neutrophils at 24 hours after operation. Neutrophils are stained with SYTOX Orange (red) and Cit-H3 (green). Arrows indicate NETs. Scale bar indicates 10 μm. (B) Quantification of the percentage of Cit-H3-positive neutrophils. (C) Levels of plasma cfDNA are measured at 24 hours after operation. (D and E) Representative immunoblots of NETs appearance (D) and quantification of the Cit-H3 levels (E) in the hippocampus at 24 hours after operation. (F) Representative immunofluorescence images of Cit-H3 (green) and MPO (red) staining with blue DAPI nuclear staining in hippocampus. Neutrophils express MPO (red) and NET forming neutrophils also express Cit-H3 (green). Cyan fluorescence represents the colocalization of Cit-H3 with DNA. The white arrows point to neutrophils with NETs and the red arrows to neutrophils without NETs. Scale bar indicates 20 μm. Higher magnification images are shown at the right row of figures-scale bar indicates 10 μm. (G) Total NETs score of each group. (H and I) Freezing to context and freezing to tone examined at 24 hours after operation. (J) The BBB permeability of hippocampus evaluated by Evans blue extravasation at 24 hours after operation. (K) Representative TUNEL (green) and DAPI (blue) immunofluorescence staining in the hippocampus. Scale bar indicates 20 μm. Higher magnification images are shown at the right row of figures-scale bar indicates 10 μm. (L) The quantitative results of the percentage of TUNEL positive area in the total area of the image (whole microscopic field) in the hippocampus. (M) Representative IBA-1 (red) and DAPI (blue) immunofluorescence staining in the hippocampus. Scale bar indicates 20 μm. Higher magnification images are shown at the right row of figures, and the scale bar indicates 10 μm. (N) The quantitative results of the percentage of IBA-1 positive area in the total area of the image (whole microscopic field) in the hippocampus. The values are presented as mean ± SD (n=6; *P<0.05, **P<0.01, ***P<0.001, ****P<0.0001, ns=not significant, one-way analysis of variance).

Given the deleterious role of neutrophils in SAE, we sought to elucidate the underlying pathogenic mechanisms. A widely described activity of neutrophils is the formation of NETs. Activated neutrophils release NETs to fight against pathogens, but NETs have also been shown to promote multi-organ damage and reduce survival in sepsis 27. In order to determine whether NETs were presented in the blood of CLP mice, citrullinated histone H3 (Cit-H3), a NET-specific biomarker 47, and SYTOX were stained on neutrophils from mouse blood collected 24 hours after CLP. The findings showed that CLP mice had considerably more Cit-H3^+^ neutrophils than sham-operated mice (Fig. 2A-B). In accordance with this, high levels of circulating DNA were discovered in the plasma from CLP mice (Fig. 2C). We then investigated whether NETs were presented in the hippocampus of CLP mice, where NETs might induce inflammatory damage and affect SAE outcomes 25. At 24 hours following CLP, a western blot analysis of the hippocampus from CLP mice revealed an elevated level of Cit-H3 (Fig. 2D-E). Immunostaining revealed that the hippocampus was extensively labeled with Cit-H3^+^ neutrophils and had higher NETs score at 24 hours after CLP (Fig. 2F-G), suggesting that NETs might play an important role during SAE. We next investigated whether *in vivo* administration of DNase I could degrade the NETs presented in blood and hippocampus. As we expected, treatment with DNase I significantly reduced the NET levels in blood and hippocampus of CLP mice (Fig. 2A-G).

Next, we sought to investigate whether the digestion of NETs was associated with improved encephalopathy induced by sepsis. FC tests showed that DNase I treatment ameliorated sepsis-induced memory impairment at 24 hours after CLP surgery (Fig. 2H-I). Compared with the vehicle controls, administration of DNase I preserved the BBB permeability in hippocampus of CLP mice (Fig. 2J). In addition, degradation of NETs attenuated neuronal apoptosis and suppressed the activation of microglia in hippocampus of CLP mice (Fig. 2K-N). TUNEL assays showed that the rate of apoptotic cells in the hippocampus was greatly increased in CLP mice, while the situation was improved in CLP mice treated with DNase I (Fig. 2K-L). The fluorescence intensity of IBA-1 was significantly stronger in the CLP mice, while DNase I treatment significantly reversed this change (Fig. 2M-N). These data indicated that DNase I could digest NETs released by neutrophils, and that neutrophil NETs might play a crucial role in SAE.

### Inhibition of GSDMD in neutrophils reduces NET release and improves SAE

**Figure 3:**
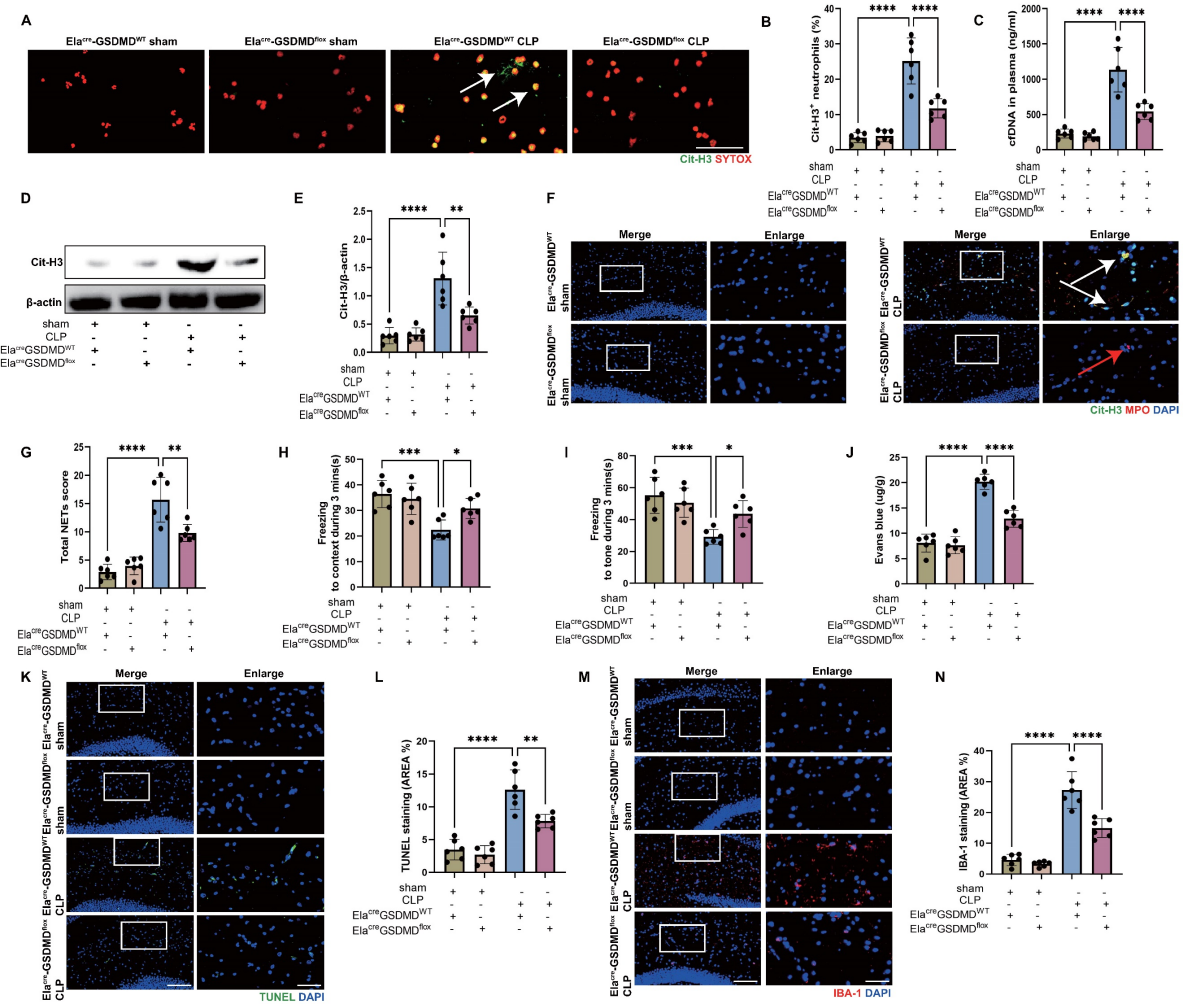
**GSDMD regulates NET release and GSDMD deficiency in neutrophils attenuates the progression of SAE.** (A) Representative immunofluorescence images of isolated peripheral blood neutrophils at 24 hours after operation. Neutrophils are stained with SYTOX Orange (red) and Cit-H3 (green). Arrows indicate NETs. Scale bar indicates 10 μm. (B) Quantification of the percentage of Cit-H3-positive neutrophils. (C) Levels of plasma cfDNA are measured at 24 hours after operation. (D and E) Representative immunoblots of NETs appearance (D) and quantification of the Cit-H3 levels (E) in the hippocampus at 24 hours after operation. (F) Representative immunofluorescence images of Cit-H3 (green) and MPO (red) staining with blue DAPI nuclear staining in hippocampus. Neutrophils express MPO (red) and NET forming neutrophils also express Cit-H3 (green). Cyan fluorescence represents the colocalization of Cit-H3 with DNA. The white arrows point to neutrophils with NETs and the red arrows to neutrophils without NETs. Scale bar indicates 20 μm. Higher magnification images are shown at the right row of figures-scale bar indicates 10 μm. (G) Total NETs score of each group. (H and I) Freezing to context and freezing to tone examined at 24 hours after operation. (J) The BBB permeability of hippocampus evaluated by Evans blue extravasation at 24 hours after operation. (K) Representative TUNEL (green) and DAPI (blue) immunofluorescence staining in the hippocampus. Scale bar indicates 20 μm. Higher magnification images are shown at the right row of figures-scale bar indicates 10 μm. (L) The quantitative results of the percentage of TUNEL positive area in the total area of the image (whole microscopic field) in the hippocampus. (M) Representative IBA-1 (red) and DAPI (blue) immunofluorescence staining in the hippocampus. Scale bar indicates 20 μm. Higher magnification images are shown at the right row of figures-scale bar indicates 10 μm. (N) The quantitative results of the percentage of IBA-1 positive area in the total area of the image (whole microscopic field) in the hippocampus. The values are presented as mean ± SD (n=6; *P<0.05, **P<0.01, ***P<0.001, ****P<0.0001, ns=not significant, one-way analysis of variance).

It is reported that GSDMD, the pore-forming protein, is active in septic neutrophils and is essential for NET release 27. Neutrophil NETs inhibition with DNase I had a protective effect on SAE (Fig. 2), so we speculated that neutrophil GSDMD knockout might have a protective effect against SAE. To test this hypothesis, we introduced neutrophil specific GSDMD conditional knockout (Ela^cre^-GSDMD^flox^) mice. First, we measured the Cit-H3^+^ neutrophils and circulating DNA in peripheral blood of CLP mice (Fig. 3A-C), indicating that gene knockout of neutrophil GSDMD reduced peripheral blood NETs levels in CLP mice. We next investigated whether neutrophil GSDMD knockout reduced NETs in the hippocampus of CLP mice. At 24 hours following CLP, a western blot analysis of hippocampus from the CLP mice revealed that gene knockout of neutrophil GSDMD reduced Cit-H3 level (Fig. 3D-E). Immunostaining also revealed that gene knockout of neutrophil GSDMD reduced Cit-H3^+^ neutrophils and NETs score in hippocampus of CLP mice (Fig. 3F-G).

Then, we explored whether neutrophil GSDMD knockout could improve SAE. We detected memory impairment with the FC tests in CLP model. These results indicated that Ela^cre^-GSDMD^WT^ CLP groups had shorter time of freezing to context and to tone compared with Ela^cre^-GSDMD^flox^ CLP groups, demonstrating that gene knockout of neutrophil GSDMD improved memory impairment (Fig. 3H-I). Next, we measured Evans blue extravasation in hippocampus, suggesting that gene knockout of neutrophil GSDMD could improve BBB integrity (Fig. 3J). In addition, gene knockout of neutrophil GSDMD suppressed neuronal apoptosis and alleviated activation of microglia in hippocampus of SAE mice (Fig. 3K-N). TUNEL assays showed that the rate of apoptotic cells in the hippocampus was greatly increased in Ela^cre^-GSDMD^WT^ mice under septic attack, while the situation was improved in Ela^cre^-GSDMD^flox^ CLP mice (Fig. 3K-L). The fluorescence intensity of IBA-1 was significantly stronger in the Ela^cre^-GSDMD^WT^ mice under septic attack, while gene knockout of neutrophil GSDMD remarkably reversed this change (Fig. 3M-N). These data indicated that GSDMD was essential for NET release by neutrophils in CLP mice, and that neutrophil GSDMD might play a crucial role in SAE.

### PD-L1 regulates GSDMD expression in neutrophils from patients with sepsis

**Figure 4:**
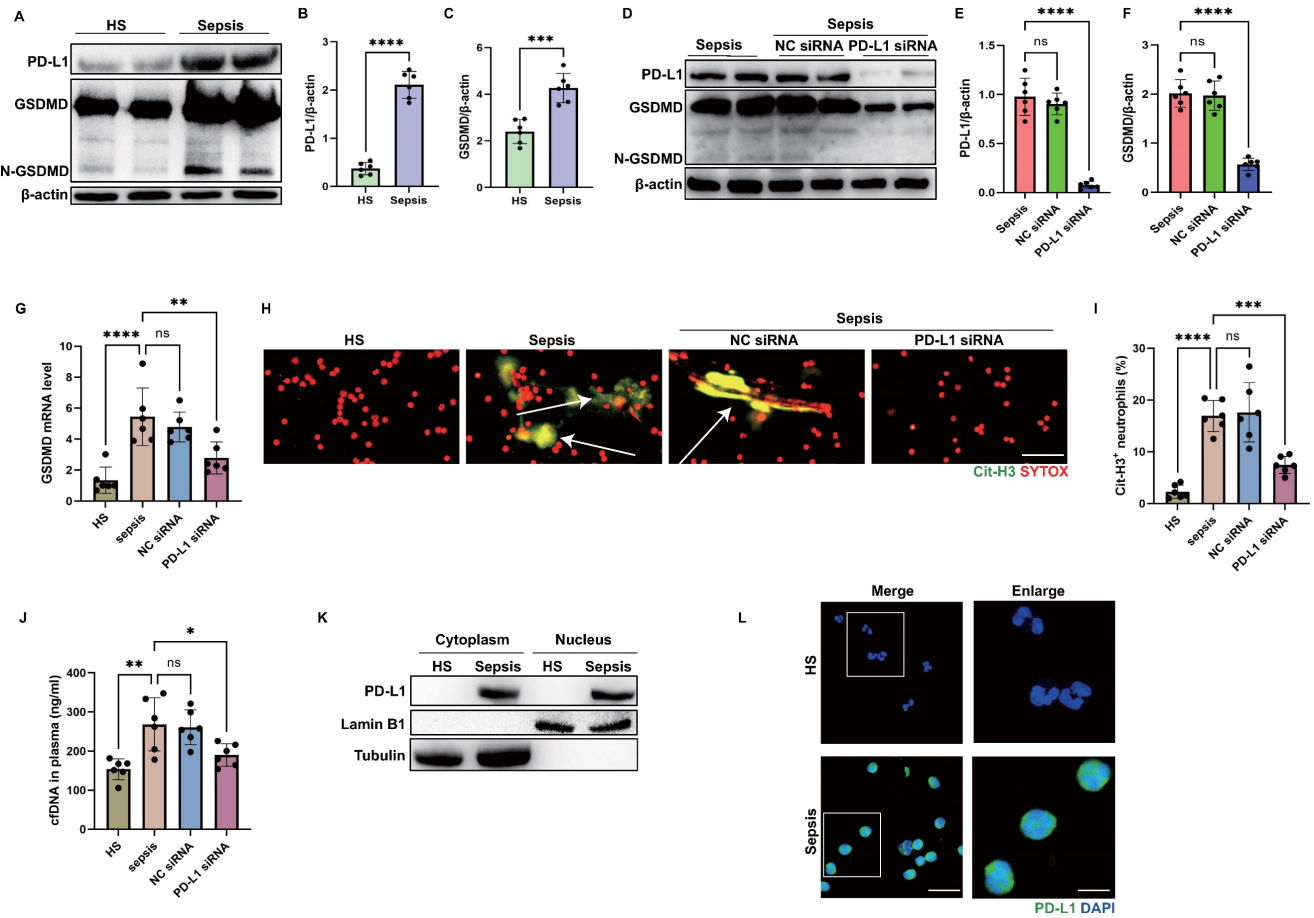
** PD-L1 can regulate GSDMD expression in septic neutrophils.** (A to C) Representative immunoblots and quantification of PD-L1 and GSDMD levels in the neutrophils from healthy subjects and septic patients. (D to F) Representative immunoblots and quantification of PD-L1 and GSDMD levels in the neutrophils from septic patients at 21 hours after PD-L1 siRNA treatment. (G) The GSDMD mRNA levels of neutrophils from septic patients at 21 hours after PD-L1 siRNA treatment. (H) Representative immunofluorescence images of isolated peripheral blood neutrophils from healthy subjects and septic patients. Neutrophils are stained with SYTOX Orange (red) and Cit-H3 (green). Arrows indicate NETs. Scale bar indicates 10 μm. (I) Quantification of the percentage of Cit-H3-positive neutrophils. (J) cfDNA levels of supernatant of cultured neutrophils are measured at 21 hours after PD-L1 siRNA treatment. (K) The nucleus is extracted from neutrophils from healthy subjects and septic patients. Representative immunoblot of PD-L1 in neutrophil nucleus. (L) Representative images of neutrophils PD-L1 from healthy subjects and septic patients by confocal microscopy. Neutrophils are stained with PD-L1 (green) and DAPI (blue). Scale bar indicates 10 μm. Higher magnification images are shown at the right row of figures-scale bar indicates 5 μm. The values are presented as mean ± SD (n=6; *P<0.05, **P<0.01, ***P<0.001, ****P<0.0001, ns=not significant, 2-tailed Student's t test for 4B, 4C, 4E, and 4F; one-way analysis of variance for 4G, 4I, 4J).

Since our previous study showed that neutrophil PD-L1 regulated NET release in acute lung injury 24, and GSDMD was demonstrated to facilitate NET release. We questioned whether there is a link between PD-L1 and GSDMD in regulating NETs. Thus, we performed a series of experiments using peripheral blood neutrophils from patients with sepsis and healthy subjects. First, we confirmed that PD-L1 and gasdermin D (GSDMD) were up-regulated in neutrophils from septic patients by immunoblot analysis (Fig. 4A-C). Next, we genetically silenced neutrophil PD-L1 expression using small interfering RNA (siRNA) to determine if the up-regulated PD-L1 was correlated with GSDMD expression. After confirming siRNA transfection successfully using western blotting, PD-L1-knockdown septic neutrophils exhibited significantly lower expression of GSDMD both at protein and mRNA levels (Fig. 4D-G).

Then we sought to investigate whether PD-L1 was correlated with NET release in septic neutrophils. Immunostaining revealed that septic neutrophils released considerable amounts of Cit-H3, but after neutrophil PD-L1 silencing, the proportion of Cit-H3^+^ neutrophils was almost diminished (Fig. 4H-I). In accordance with this, high levels of circulating DNA were discovered in the supernatant of cultured neutrophils from septic patients, while PD-L1 siRNA treatment attenuated the release of DNA in the supernatant of neutrophils significantly (Fig. 4J). These two parts indicated that neutrophil PD-L1 silencing could reduce NET release in septic neutrophils.

Since genetic silence of PD-L1 resulted in reduced expression of GSDMD at both of the mRNA and protein levels, we questioned whether PD-L1 was present in the nuclei so as to regulate the GSDMD mRNA transcription. We extracted the nuclei of neutrophils and found that PD-L1 was also abundant in the nuclei of neutrophils from septic patients, but not in those from healthy subjects (Fig. 4K). This was also validated by confocal microscopy analysis using anti-PD-L1 antibody (Fig. 4L). These results dedicated that PD-L1 might regulate GSDMD mRNA transcription to regulate NET release in septic neutrophils, which might be associated with PD-L1 translocating into nuclei of septic neutrophils.

### Sepsis induces nPD-L1 translocation through p-Y705-Stat3 which transcriptionally activates GSDMD expression in neutrophils

**Figure 5:**
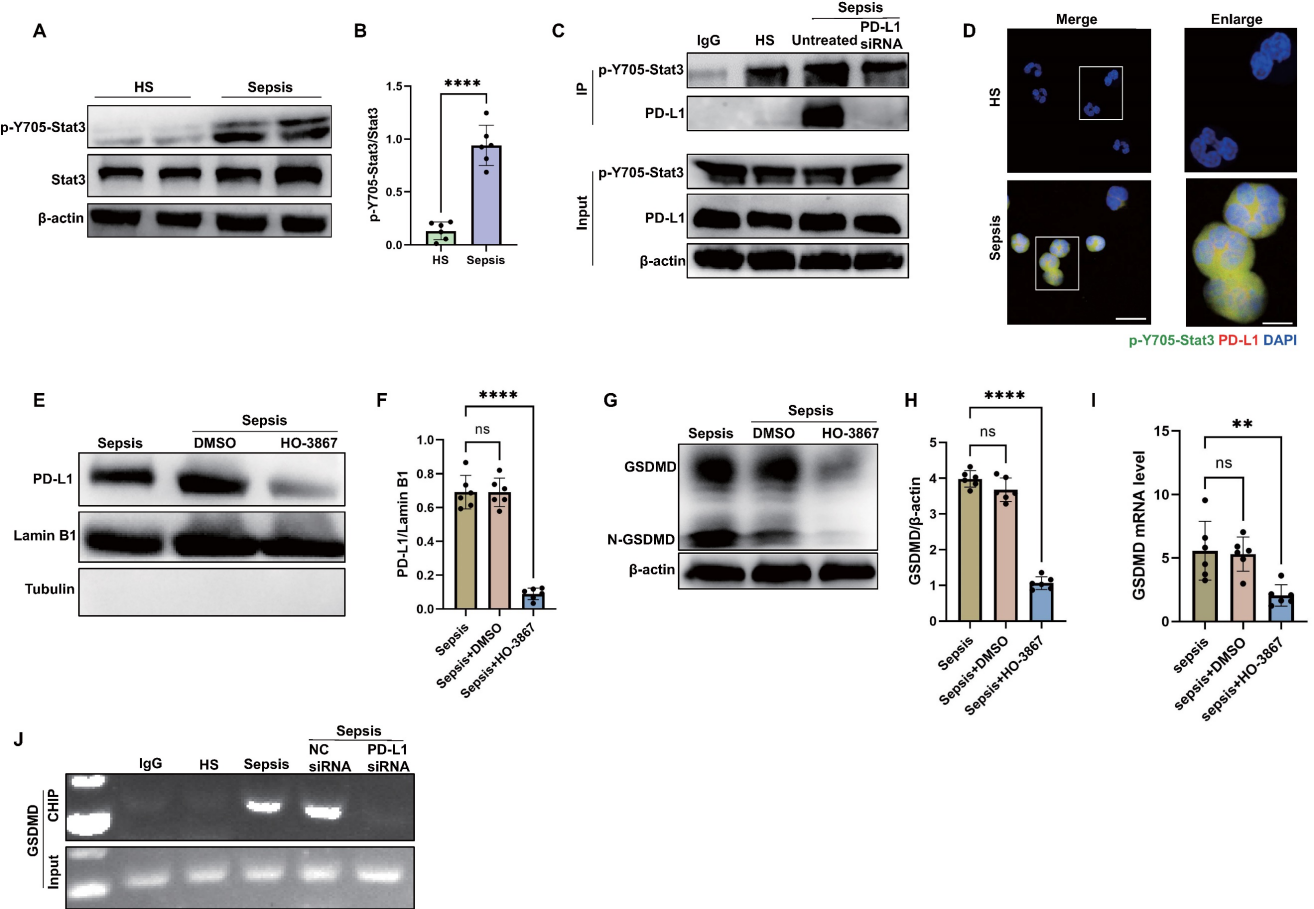
** nPD-L1 forms a complex with p-Y705-Stat3 to transcriptionally activate GSDMD expression in neutrophils from septic patients.** (A and B) Representative immunoblots and quantification of p-Y705-Stat3 level in the neutrophils from healthy subjects and sepsis patients. (C) Immunoprecipitation (IP) and western blot analysis of the PD-L1/p-Y705-Stat3 interaction in neutrophils from healthy subjects and septic patients. (D) Representative images of neutrophils PD-L1/p-Y705-Stat3 interaction from healthy subjects and septic patients by confocal microscopy. Neutrophils are stained with p-Y705-Stat3 (green), PD-L1 (red) and DAPI (blue). Scale bar indicates 10 μm. Higher magnification images are shown at the right row of figures, and the scale bar indicates 5 μm. (E and F) The nucleus is extracted from septic neutrophils at 24 hours after treating with DMSO or inhibitor HO-3867 (20 μM). Representative immunoblots and quantification of PD-L1 level in neutrophil nucleus. (G and H) Representative immunoblots and quantification of GSDMD level in the neutrophils from septic patients at 24 hours after treating with DMSO or inhibitor HO-3867 (20 μM). (I) The GSDMD mRNA levels of neutrophils from septic patients at 24 hours after treating with DMSO or inhibitor HO-3867 (20 μM). (J) Sequential ChIP-PCR analysis of the interactions between p-Y705-Stat3 and the GSDMD promoter in septic neutrophils. The values are presented as mean ± SD (n=6; **P<0.01, ****P<0.0001, ns=not significant, 2-tailed Student's t test for 5B; one-way analysis of variance for 5F, 5H, and 5I).

Then, we investigated how PD-L1 translocated into the nuclei of septic neutrophils. Given that Stat3-Y705 is phosphorylated (p-Y705-Stat3) in response to sepsis, and p-Y705-Stat3 can translocate to the nucleus or mitochondria to regulate the expression of target genes 48 and it has been proposed as a therapeutic target for sepsis through regulating inflammation 49, 50, we examined the interaction between p-Y705-Stat3 and PD-L1 in neutrophils from septic patients. As shown by the western blot, Stat3-Y705 phosphorylation was enhanced in septic neutrophils (Fig. 5A-B). The results by immunoprecipitation and confocal microscopy indicated that PD-L1 interacted physically with p-Y705-Stat3 (Fig. 5C-D). In addition, the inhibition of p-Y705-Stat3 by its inhibitor HO-3867 suppressed the nPD-L1 translocation and also reduced GSDMD protein and mRNA expression in septic neutrophils (Fig. 5E-I), indicating that p-Y705-Stat3 was necessary for nPD-L1 translocation and GSDMD expression. In PD-L1 knockdown neutrophils, we found that GSDMD protein and mRNA expression were dramatically suppressed (Fig. 4D-G). These data indicated that both nPD-L1 and p-Y705-Stat3 were essential for GSDMD expression in septic neutrophils.

We used the bioinformatics tool Jaspar to research for Stat3 binding sites located within the promoter of human GSDMD gene (Table S2), suggesting that p-Y705-Stat3 might be a transcription factor for human GSDMD. Then, sequential chromatin immunoprecipitation (ChIP)-PCR verified that p-Y705-Stat3 could bind to the GSDMD promoter (Fig. 5J). Together, these results suggested that PD-L1 might interact with p-Y705-Stat3 to facilitate the translocation of PD-L1/p-Y705-Stat3 complex and initiate the transcription of GSDMD mRNA in neutrophils during sepsis.

### Inhibition of PD-L1 on neutrophils reduces NET release and improves SAE

**Figure 6:**
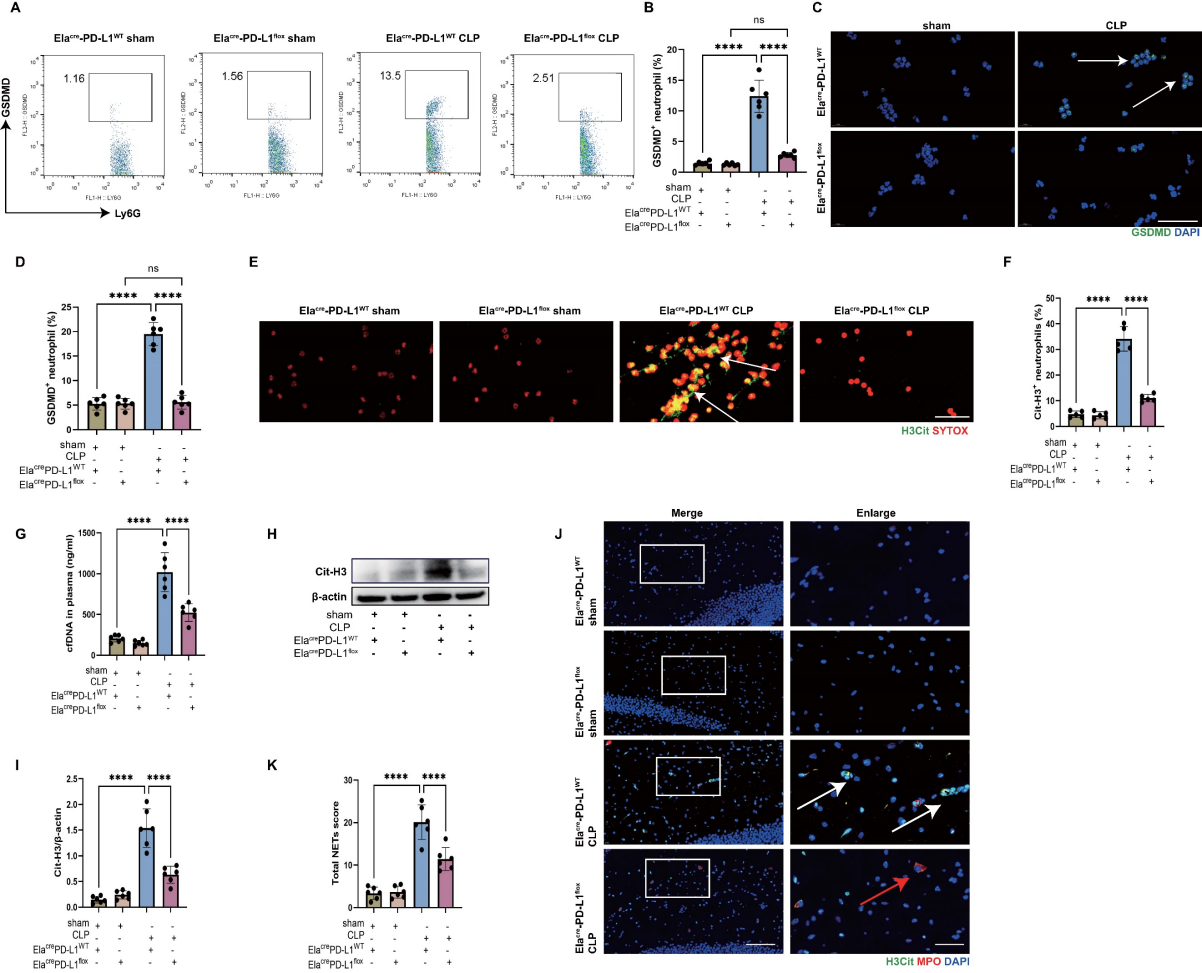
** Genetic deletion of neutrophil PD-L1 reduces the expression of GSDMD in neutrophils *in vivo* and attenuates the release of NETs in CLP mice.** (A and B) Representative FACS plots and quantification of GSDMD^+^ neutrophils measured by flow cytometry in blood at 24 hours after operation. (C) Representative immunofluorescence images of isolated peripheral blood neutrophils at 24 hours after operation. Neutrophils were stained with GSDMD (green) and DAPI (blue). Scale bar indicates 20 μm. Arrows indicate GSDMD^+^ neutrophils. (D) Quantification of the percentage of GSDMD-positive neutrophils. (E) Representative immunofluorescence images of isolated peripheral blood neutrophils at 24 hours after operation. Neutrophils are stained with SYTOX Orange (red) and Cit-H3 (green). Arrows indicate NETs. Scale bar indicates 10 μm. (F) Quantification of the percentage of Cit-H3-positive neutrophils. (G) Levels of plasma cfDNA are measured at 24 hours after sham or CLP surgery. (H and I) Representative immunoblots of NETs appearance (H) and quantification of the Cit-H3 levels (I) in the hippocampus at 24 hours after operation. (J) Representative immunofluorescence images of Cit-H3 (green) and MPO (red) staining with blue DAPI nuclear staining in hippocampus. Neutrophils express MPO (red) and NET forming neutrophils also express Cit-H3 (green). Cyan fluorescence represents the colocalization of Cit-H3 with DNA. The white arrows point to neutrophils with NETs and the red arrows to neutrophils without NETs. The scale bar indicates 20 μm. Higher magnification images are shown at the right row of figures-scale bar indicates 10 μm. (K) Total NETs score of each group. The values are presented as mean ± SD (n=6; ****P<0.0001, ns=not significant, one-way analysis of variance).

Finally, an *in vivo* experiment was performed to observe the role of neutrophil PD-L1 in NET release and development of SAE with neutrophil specific PD-L1 conditional knockout (Ela^cre^-PD-L1^flox^) mice. First, we confirmed the GSDMD expression in neutrophils of peripheral blood in CLP-induced sepsis model using Ela^cre^-PD-L1^flox^ mice. The results by flow cytometry and immunostaining showed that peripheral blood neutrophils expressed less GSDMD in Ela^cre^-PD-L1^flox^ CLP mice than in Ela^cre^-PD-L1^WT^ CLP mice (Fig. 6A-D). These data provided strong *in vivo* evidence that PD-L1 was important for GSDMD expression in septic neutrophils.

We then measured the Cit-H3^+^ neutrophils and circulating DNA in peripheral blood of CLP mice, which were less in Ela^cre^-PD-L1^flox^ mice (Fig. 6E-G), indicating that gene knockout of neutrophil PD-L1 reduced peripheral blood NET levels in CLP mice. We next investigated whether neutrophil PD-L1 inhibition reduced NETs in the hippocampus of CLP mice. At 24 hours following CLP, a western blot analysis of the CLP mice hippocampus revealed that gene knockout of neutrophil PD-L1 reduced Cit-H3 level (Fig. 6H-I). Immunostaining revealed that gene knockout of neutrophil PD-L1 reduced Cit-H3^+^ neutrophils and NETs score in hippocampus of CLP mice (Fig. 6J-K). These data demonstrated that knockout of neutrophil PD-L1 reduced NETs levels in peripheral blood and hippocampus in CLP mice.

**Figure 7:**
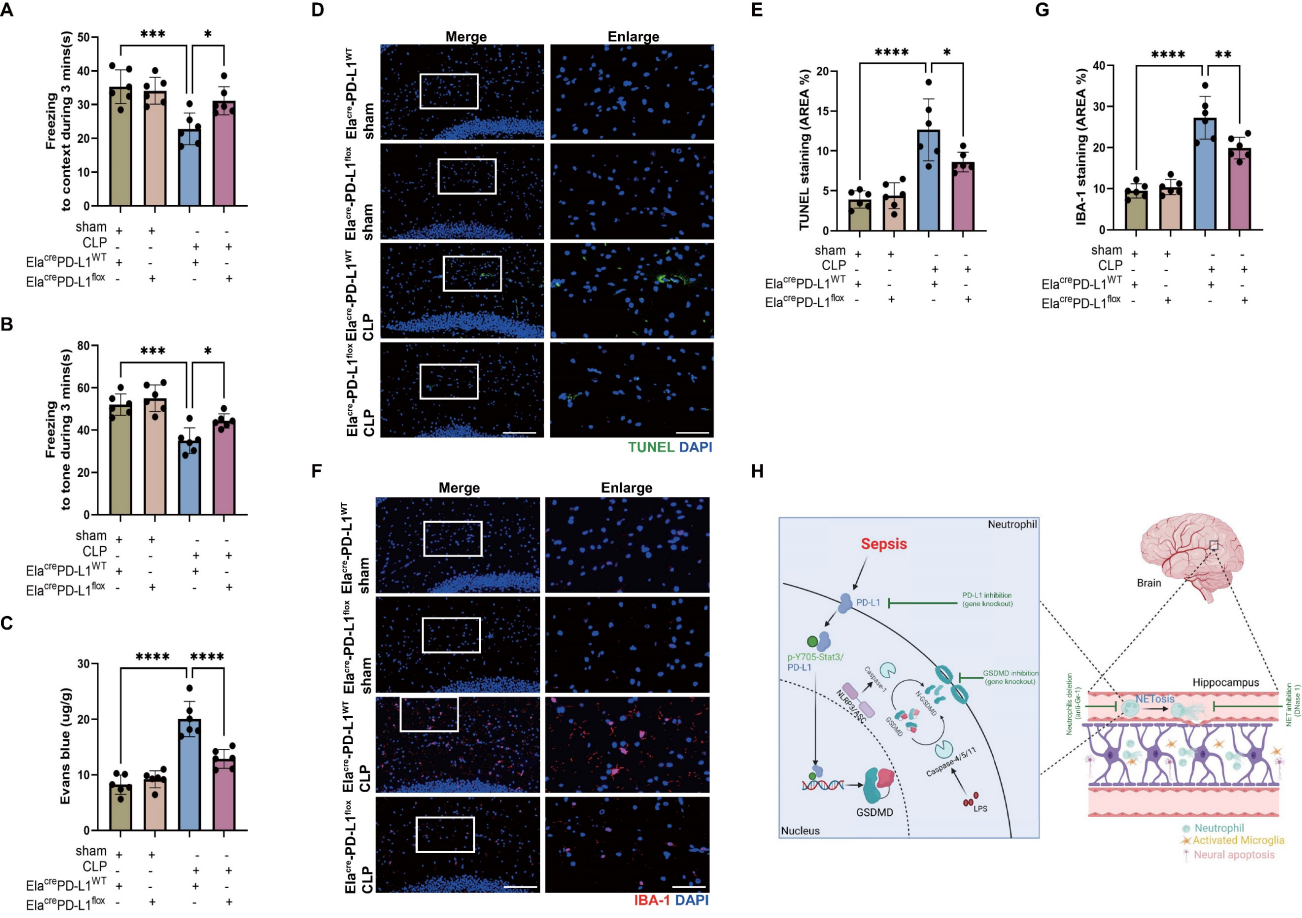
** Genetic knockout of Neutrophil PD-L1 attenuates the progression of SAE.** (A and B) Freezing to context and freezing to tone examined at 24 hours after operation. (C) The BBB permeability of hippocampus evaluated by Evans blue extravasation at 24 hours after operation. (D) Representative TUNEL (green) and DAPI (blue) immunofluorescence staining in the hippocampus. Scale bar indicates 20 μm. Higher magnification images are shown at the right row of figures-scale bar indicates 10 μm. (E) The quantitative results of the percentage of TUNEL positive area in the total area of the image (whole microscopic field) in the hippocampus. (F) Representative IBA-1 (red) and DAPI (blue) immunofluorescence staining in the hippocampus. Scale bar indicates 20 μm. Higher magnification images are shown at the right row of figures-scale bar indicates 10 μm. (G) The quantitative results of the percentage of IBA-1 positive area in the total area of the image (whole microscopic field) in the hippocampus. (H) Schematic illustration of the main findings: Sepsis-induced NETosis contributes to hippocampus-dependent memory impairment, and increases BBB permeability, neuronal apoptosis, and microglia activation in the hippocampus region. The NET release is promoted by the cleavage of GSDMD, which is transcriptionally regulated by the nuclear translocation of a PD-L1/p-Y705-Stat3 complex. Together, PD-L1/Stat3/GSDMD is essential for NET production and development of sepsis-associated encephalopathy. The values are presented as mean ± SD (n=6; *P<0.05, **P<0.01, ***P<0.001, ****P<0.0001, one-way analysis of variance).

We have shown that reducing NETs with DNase I and neutrophil GSDMD knockout improved SAE (Fig. 2 and Fig. 3), and we then explored whether inhibition of neutrophil PD-L1 could improve SAE. FC tests indicated that Ela^cre^-PD-L1^WT^ CLP groups had shorter time of freezing to context and to tone compared with Ela^cre^-PD-L1^flox^ CLP groups, demonstrating that gene knockout of neutrophil PD-L1 improved memory impairment (Fig. 7A-B). Next, we measured Evans blue extravasation, suggesting that knockout of neutrophil PD-L1 could improve BBB integrity (Fig. 7C). In addition, gene knockout of neutrophil PD-L1 suppressed neuronal apoptosis and alleviated activation of microglia in hippocampus of CLP mice (Fig. 7D-G). TUNEL assays showed that the rate of apoptotic cells in the hippocampus was greatly increased in Ela^cre^-PD-L1^WT^ mice under septic attack, while the situation was improved in Ela^cre^-PD-L1^flox^ CLP mice (Fig. 7D-E). The fluorescence intensity of IBA-1 was significantly stronger in the Ela^cre^-PD-L1^WT^ mice under septic attack, while gene knockout of neutrophil PD-L1 significantly reversed this change (Fig. 7F-G). Together, above data indicated that PD-L1 was necessary for NET release by regulating neutrophil GSDMD expression in septic mice, and that neutrophil PD-L1 might play a crucial role in SAE.

## Discussion

This study demonstrated that neutrophils and NETs were indispensable in the pathogenesis of SAE. Neutrophil GSDMD was essential for NET release in sepsis and promoted subsequent development of SAE. Importantly, the elevation of GSDMD was dependent on the nuclear translocation of PD-L1/p-Y705-Stat3 complex, which was responsible for the transcription of GSDMD mRNA in septic neutrophils. Finally, *in vivo* knockout of PD-L1 in neutrophils prevented the release of NETs in sepsis and attenuated the development of SAE (summarized in Fig. 7H).

Sepsis is defined as an infection-induced abnormal immune response leading to life-threatening organ dysfunction, and usually leads to diffuse brain dysfunction, which is referred to as SAE 10. The pathogenesis of SAE is still unclear. In the present study, we used the CLP method, a classical way to establish an animal model of sepsis 37, to explore the potential pathogenesis of SAE. CLP induced a hippocampus-dependent memory impairment, and increased BBB permeability, neuronal apoptosis, and microglia activation in hippocampus, which was consistent with previous reports by us and others 40, 51-53. Our data showed that depletion of neutrophils with anti-Gr-1 antibody and degradation of NETs with DNase I reversed the sepsis-induced changes in the hippocampus, suggesting that neutrophils and NETs might play a crucial role in SAE.

Neutrophils serve as the first line of defense against pathogens in the innate immune responses and play an important defense function early after the onset of sepsis. However, neutrophils also undergo reprogramming, as evidenced by impaired chemotaxis, abnormal accumulation and decreased potency 54. Our data showed that neutrophils accumulated in hippocampus of CLP mice, which was corresponded to another study 44, suggesting that neutrophils may contribute to the development of SAE. Indeed, the results suggested that neutrophil depletion attenuated hippocampus-dependent memory impairment and inflammatory damages of hippocampus. One of the mechanisms underlying in the bactericidal activity of neutrophil is the release of NETs, which are extracellular DNA web-like structure containing histones and cytotoxic enzymes 14. Despite this bactericidal effect of NETs, there is growing evidence that NETs also mediate tissue damage under various inflammatory conditions, including sepsis 15-18. We demonstrated that NETs were abundant in the blood and hippocampus of CLP mice, suggesting that NETs may be important to the development of SAE. As we expected, the sepsis-induced changes in the hippocampus are mitigated by the degradation of NETs with DNase I. These findings indicated that neutrophils might exert a detrimental role to promote SAE development through releasing NETs. It was also reported that the MPO activity and the release of ROS and inflammatory cytokines played an important role in the neuroinflammation of septic mice 55. Our study demonstrated that NETs were an important mediator of SAE, but did not exclude the potential effect of other neutrophil activities on SAE.

Sepsis-induced BBB disruption is considered to be an important step in the development of SAE 53. Under normal conditions, an intact BBB is essential for normal neuronal function and protection of the CNS from toxins, pathogens, inflammation, injury and diseases 56. However, once the BBB is disrupted by sepsis, neutrophils can infiltrate into the CNS to produce a series of adverse factors 57, 58. In addition to promoting neuronal damage 59, activated neutrophils can also lead to activation of microglia 57. NETs contain a variety of cytotoxic proteases, such as histone, MPO, elastase, etc., which directly induce endothelial cell damage and increase vascular permeability 60. We observed that neutrophils isolated from CLP mice and septic patients exhibited a greater tendency to form spontaneous NETs, which also can be presented in the hippocampus of CLP mice. Multiple stimuli in sepsis activate neutrophils to release excessive NETs in the vasculature and parenchyma, consistent with elevated circulating DNA. NETs digestion with DNase I significantly reduces BBB damage in hippocampus of CLP mice. Therefore, our data showed that NETs were able to damage the BBB of hippocampus in a murine sepsis model, and subsequently neutrophils were able to enter the hippocampus to produce NETs.

Neuronal apoptosis and microglia activation are also important in the pathogenesis of SAE 52. NETs are cytotoxic and can cause direct damage to neurons 43. Our data indicated that degradation of NETs with DNase I significantly reduced the number of TUNEL-positive cells in hippocampus of CLP mice, suggesting that DNase I attenuates neuronal apoptosis in hippocampus. Under inflammatory conditions, NETs released from neutrophils can cause macrophages to undergo polarization toward the M1 phenotype 61. We found that degradation of NETs inhibited the expression of IBA-1, a marker of microglia activation. Therefore, these results suggested that NETs might induce neuronal apoptosis and microglia activation in hippocampus to promote the development of SAE.

The importance of GSDMD in the formation of neutrophil NETs has been confirmed by several previous studies 27-29. Human caspase-4/5 or mouse caspase-11 cleaves GSDMD to form pores in the granular membrane of neutrophils, thus releasing NE and MPO at the early stage of NET formation. The late stage of NET formation also requires the activation of GSDMD, which is responsible for creating pores on the cell membrane to allow NET extrusion 27. A recent study showed that neutrophil GSDMD inhibition by genetic deletion or GSDMD inhibitor disulfiram efficiently abrogated NETosis and prevented vital multi-organ dysfunction and improved survival 27. We demonstrated that genetic deletion of GSDMD in neutrophils efficiently reduced NET release and attenuated the progression of SAE in CLP-induced sepsis model.

Conventionally, various microbial signals are detected by typical inflammatory vesicle sensors and activate caspase-1 via ASC or NLRC4 adapters. Caspase-4/5/11 are activated by direct binding to LPS. Activated caspase-1 and caspase-11/4/5 cleave the GSDMD to form the N-GSDMD, which forms pores in the cell membrane and releases inflammatory factors or NETs 62. But most of the studies were focused on the activation of GSDMD, the transcriptional regulation of GSDMD was seldom reported. Our data demonstrated that changes in GSDMD expression occurred not only at the level of N-GSDMD but also at the GSDMD transcriptional level according to the increased mRNA of GSDMD in septic neutrophils. Although advances in our understanding of the molecular mechanisms leading to GSDMD pore formation, little is known about the transcriptional mechanisms underlying GSDMD expression in neutrophils. Liu et al. demonstrated that in adipocytes NF-κB enhances GSDMD transcription by binding to the GSDMD promoter 63. Kayagaki et al. demonstrated that IRF2 can directly transcriptionally induce GSDMD expression in macrophages and endothelial cells 64. However, in septic neutrophils, the transcriptional regulation of GSDMD is unclear. In this study, the role of PD-L1 and Stat3 in the transcriptional regulation of GSDMD in neutrophils has been investigated.

To date, most PD-L1 studies have focused on its immune-checkpoint function. Here, we identified a non-immune-checkpoint function by which PD-L1 regulated GSDMD expression to promote NET release in septic neutrophils. *In vitro*, GSDMD expression in septic neutrophils was inhibited after PD-L1 siRNA transfection. In addition, *in vivo* evidence proved that neutrophils expressed less GSDMD in Ela^cre^-PD-L1^flox^ mice than in Ela^cre^-PD-L1^WT^ mice under sepsis. Interestingly, it has been reported that in tumor cells, PD-L1 can undergo nuclear translocation under hypoxic conditions 65. Our data suggested that PD-L1 could also translocated into the nuclei of neutrophils during sepsis.

During sepsis, Stat3 is activated by multiple signaling pathways and plays a key role in transcriptional regulation of the correlated gene through phosphorylation on Tyr705 residue 48. In human septic neutrophils, we found that PD-L1 interacted with p-Y705-Stat3 and translocated into the nuclei. GSDMD expression was inhibited in the absence of PD-L1 or p-Y705-Stat3. The ChIP assay further confirmed that p-Y705-Stat3 was a transcriptional factor for the initiation of GSDMD transcription. These data suggested that the nPD-L1/p-Y705-Stat3 complex might be essential for transcriptionally promoting GSDMD expression.

Our study might propose some therapeutic implications for SAE. We demonstrated that inhibition of neutrophil PD-L1 or GSDMD reduced the release of NETs and improved SAE, including improving hippocampus-dependent memory impairment and attenuating BBB disruption, neuronal apoptosis and microglia activation. Therefore, neutrophil PD-L1 and GSDMD may be two potential targets for the treatment of SAE. We and others have shown that anti-PD-L1 antibodies or peptides can improve survival in septic mice 30, 66, and our present study provided further evidences for the clinical use of anti-PD-L1 in treating sepsis. As an inhibitor of GSDMD, disulfiram could inhibit NET release during sepsis 27, and had been involved in a clinical trial to treat SARS-CoV-2 infection 67. It might also be valuable to investigate the therapeutic role of disulfiram in treating SAE.

In summary, our results have shown that neutrophils and NET production are involved in the pathogenesis of encephalopathy induced by sepsis. PD-L1-mediated GSDMD upregulation is responsible for the NET release and the development of SAE. Neutrophil PD-L1 can translocate to the nucleus with the assistance of p-Y705-Stat3 to form the nPD-L1/p-Y705-Stat3 complex and promote GSDMD mRNA transcription. PD-L1 and GSDMD may be new therapeutic targets against SAE.

## Supplementary Material

Supplementary tables.

## Abbreviations

BBB: blood-brain barrier
Cit-H3: citrullinated histone H3
CLP: cecal ligation and puncture
CNS: central nervous system
FC: fear conditioning
GSDMD: gasdermin D
MPO: myeloperoxidase
NE: neutrophil elastase
NETs: neutrophil extracellular traps
PD-L1: programmed death ligand 1
SAE: sepsis-associated encephalopathy
WB: western blot
