# Supplementary material for: PD-L1 promotes GSDMD-mediated NET release by maintaining the transcriptional activity of Stat3 in sepsis-associated encephalopathy
Source: Int J Biol Sci. 2023 Feb 27;19(5):1413–29. doi: 10.7150/ijbs.79913 (PMC10086742; doi:10.7150/ijbs.79913)
Supplement: Supplementary file 1 — Supplementary tables. [file ijbsv19p1413s1.pdf]

Title: PD-L1 promotes GSDMD-mediated NET release by maintaining the transcriptional activity of Stat3 in sepsis-associated encephalopathy

Author: Cheng-long Zhu[1†], Jian Xie[1†], Qiang Liu[1,2†], Yi Wang[1], Hui-ru Li[1,3], Chang-meng Yu[1,2], Peng Li[1], Xiao-ming Deng[1,2,3]*, Jin-jun Bian[1]*, Jia-feng Wang[1]*

Affiliations:

[1] Faculty of Anesthesiology, Changhai Hospital, Naval Medical University, Shanghai, People's Republic of China

[2] Jiangsu Province Key Laboratory of Anesthesiology, Xuzhou Medical University, Xuzhou, Jiangsu Province, People's Republic of China

[3] Faculty of Anesthesiology, Weifang Medical University, Weifang, Shandong Province, People's Republic of China

***Corresponding Authors**: Jia-feng Wang, M.D., Ph.D., Faculty of Anesthesiology, Changhai Hospital, the Naval Medical University, 168 Changhai Road, Yangpu District, Shanghai 200433, People's Republic of China. Email: jfwang@smmu.edu.cn. Jin-jun Bian, M.D., Ph.D., Faculty of Anesthesiology, Changhai Hospital, the Naval Medical University, 168 Changhai Road, Yangpu District, Shanghai 200433, People's Republic of China. Email: jinjunbicu@163.com. Xiao-ming Deng, M.D., Ph.D., Faculty of Anesthesiology, Changhai Hospital, the Naval Medical University, 168 Changhai Road,

Yangpu District, Shanghai 200433, People's Republic of China. Email: dengphd@smmu.edu.cn.

Table S1

The primers for PCR or RT-PCR in this study.

PCR	GSDMD-Forward: 5'-CTCGCCGACTTCCGTAAACT-3'
	GSDMD-Reverse: 5'-CTTGACCTTGCTATCCACCC-3'
RT-PCR	GSDMD-Forward: 5'-GTGTGTCAACCTGTCTATCAAGG-3'
	GSDMD-Reverse: 5'-CATGGCATCGTAGAAGTGGAAG-3'
RT-PCR	β-actin-Forward: 5'-GCACAGAGCCTCGCCTT-3'
	β-actin-Reverse: 5'-GTTGTCGACGACGAGCG-3'

Table S2

JASPAR analysis results for Stat3 binding sites located within the promoter of human GSDMD gene.

Relative Score	Start	End	Strand	Predicted Sequence
0.982	552	562	-	TTTCCGGGAAG
0.957	694	704	+	TTTCCAGGAAT
0.955	694	704	-	ATTCCTGGAAA
